# Barriers to implementation of smoking cessation support among healthcare professionals in the secondary healthcare sector: A qualitative and quantitative evaluation

**DOI:** 10.18332/tpc/183775

**Published:** 2024-02-22

**Authors:** Camilla Uhre Jørgensen, Anders Løkke, Peter Hjorth, Charlotta Pisinger, Ingeborg Farver-Vestergaard

**Affiliations:** 1 Department of Medicine, Lillebaelt Hospital, Vejle, Denmark; 2 Department of Regional Health Research, University of Southern Denmark, Odense, Denmark; 3 Psychiatric Department, Mental Health Services, University Hospital of Southern Denmark, Odense, Denmark; 4 Center of Clinical Research and Prevention, Bispebjerg-Frederiksberg University Hospital, Copenhagen, Denmark; 5 National Institute of Public Health, University of Southern Denmark, Odense, Denmark

**Keywords:** addiction, health services, mixed-methods, hospital care

## Abstract

**INTRODUCTION:**

Smoking cessation support (SCS) in the hospital is essential; patients often struggle to maintain quit attempts, which necessitates assistance from healthcare professionals (HCPs). However, unknown barriers can obstruct the implementation of SCS in hospitals. This study aims to uncover barriers to the implementation of SCS in psychiatric, somatic, inpatient, and outpatient hospital settings.

**METHODS:**

In the period from June to September 2021, HCPs in a large secondary care hospital in the Region of Southern Denmark completed an online, cross-sectional study, providing sociodemographic details and listing potential barriers to SCS. They also shared additional barriers in the form of free-text responses. Descriptive statistics and thematic analysis of free-text responses were performed.

**RESULTS:**

Of 1645 HCPs surveyed, 409 elaborated their response in the free-text field assessing unlisted barriers. Top listed barriers, reported by more than one-third of participants, included: ‘lack of time’ (45.1%), ‘lack of patient motivation’ (34.3%), and ‘insufficient knowledge on how to support’ (32.2%). Free-text responses revealed three barrier-related, which we grouped under the themes of: ‘Concerned about the patient’, ‘Not part of my job’, and ‘Inappropriate setting’.

**CONCLUSIONS:**

This quantitative and qualitative study identifies barriers to SCS on multiple levels in the hospital setting, i.e. on the patient, provider, and organizational levels. These results can inform healthcare organizations and professionals in the implementation of SCS in routine hospital care.

## INTRODUCTION

Tobacco smoking is the most significant preventable risk factor for the development and progression of mental and somatic diseases^1,2^. Approximately ten years of life reduction is documented among people who smoke compared to those who have never smoked^3^. The health benefits of discontinuing smoking are well established, and the effects of smoking cessation are rapidly seen^3^. Moreover, smoking cessation support (SCS) has shown to be a cost-effective intervention^4^. For the reasons stated above, SCS should be considered among the most important tasks of healthcare professionals (HCPs).

Receiving a diagnosis of and undergoing treatment for severe illness have been described as ‘teachable moments’ where patients’ motivation to quit smoking can be at a peak^5^. For example, one study describes that almost one-third of smokers with lung cancer quit around the time of diagnosis and that first-time malignancy patients are twice as likely to quit smoking^6^. Another study reports an incident diagnosis of heart disease and asthma related to a higher probability of quitting^7^.

SCS can be delivered in various doses and formats in accordance with the specific setting^8,9^. To increase the chances of long-term smoking abstinence, recent evidence recommends a combination of professional behavioral support and pharmacological treatment, e.g. nicotine replacement therapy, varenicline, bupropion, and/or cytisine^10-12^. Smoking cessation programs of a longer duration and with multiple counseling sessions are often delivered in specialized care units.

SCS in a hospital setting can consist of giving brief, 3–5 minute advice, e.g. using the 5As support framework (Ask, Advise, Assess motivation, Assist initiation, Arrange follow-up)^13^ or the Very Brief Advice (VBA) method (Ask if the patient is currently smoking; Inform the patient about the most effective way of quitting; Refer the patient to an evidence-based smoking cessation program) to guide referral to a specialized smoking cessation program^14^. The provision of SCS during inpatient and outpatient visits should, therefore, be considered an integral part of hospital care. Nonetheless, there is limited implementation of SCS in healthcare settings, a service that is needed, given that many patients fail to maintain their attempt to quit smoking and hence need HCP support^7^. In Denmark, where the present study was conducted, national policy states that smoking cessation programs should be delivered by the municipalities. Patients are referred to these programs via their general practitioner or HCPs at the hospital, using the VBA method for referral.

We have previously shown that 54.0% of a sample of hospital-based HCPs in Denmark report that they never or rarely assess patients’ readiness to quit smoking^15^. It appears that HCPs face challenges in implementing SCS in specific somatic and psychiatric hospital settings. According to the Consolidated Framework for Implementation Research (CFIR)^16^, implementation effectiveness is impacted by the intervention that is being implemented, the internal and external setting, the individuals involved, and the process by which implementation is accomplished. These complex and interrelated factors should be investigated and understood to facilitate the implementation of SCS. Previously identified barriers to SCS among HCPs include lack of knowledge, lack of time, and a lack of perceived patient motivation to quit smoking^17^.

Therefore, in the present study, we seek to explore barriers to the implementation of SCS among HCPs across both psychiatric and somatic, as well as inpatient and outpatient, hospital settings.

## METHODS

The present study is part of a larger, cross-sectional survey^15^ performed by researchers from the Department of Medicine, Lillebaelt Hospital, Vejle, and the Psychiatric Department, Mental Health Services, Vejle.

### Data collection and participants

An electronic survey was designed using the Danish web-based survey system *SurveyXact* and distributed among healthcare professionals (HCPs) employed at hospitals in the Southern Region of Denmark, which covers four geographical locations: Vejle, Kolding, Middelfart, and Svendborg. HCPs included doctors, nurses, healthcare assistants, social workers, psychologists, physiotherapists, occupational therapists, pedagogues, and students. Participation in the survey was voluntary and anonymous. According to Danish legislation (the Act on Research Ethics Review of Health Research Projects §14, Sect. 2), studies that collect data exclusively via questionnaires do not need approval from an ethics committee. The hospital management approved study procedures, and the processing of personal data was approved by the Region of Southern Denmark and listed in the internal records of the region prior to the initiation of data collection (no. 21/16770). As participation in the study was anonymous, participants could not give their named consent. Because of the retrospective nature of the study, informed consent was waived by the Research Support Office, Lillebaelt Hospital, Vejle, which administered the internal record. All study procedures were performed in accordance with relevant guidelines and regulations. The survey consisted of 39 items, including sociodemographic and work-related variables, the practices of SCS, and barriers to SCS. The questionnaire was developed among a group of researchers and clinicians with extensive experience in SCS. While the present study focuses only on the barriers to SCS, results in relation to SCS practices have been published elsewhere^15^.

### Quantitative evaluation

In the survey, participants were presented with a list of potential barriers to SCS in their current work setting (e.g. ‘lack of incentive’, ‘negative experience in the past’, and ‘smoking viewed as a coping mechanism for patients’). The list was developed on the basis of the results of a systematic review of qualitative studies that investigated barriers to SCS in hospital settings^17^. There was no limit to the number of barriers each participant could select.

We calculated the frequency by which the participants reported each barrier and presented frequencies of all individual barriers in rank order using the IBM SPSS Statistics Version 28.0.0.0 software.

### Qualitative evaluation

In a free-text field in the survey, participants were encouraged to elaborate on any factors that they considered hindered them from implementing SCS.

Free-text responses were evaluated using the thematic analysis framework^18^. To understand the dataset, the analysis began with a detail-oriented reading of the answers by gradually identifying and defining codes. Data coding was based on an inductive approach, which was primarily undertaken by the first author (CUJ) and discussed continuously with co-authors IFV, PH, and AL until subthemes and final themes were established. Please find an overview of codes and themes in the Supplementary file. During the analytical process, a reflective logbook was kept using the NVivo software (NVivo, Version 11.3.0.773; QSR, 1999–2016).

## RESULTS

**Figure 1:**
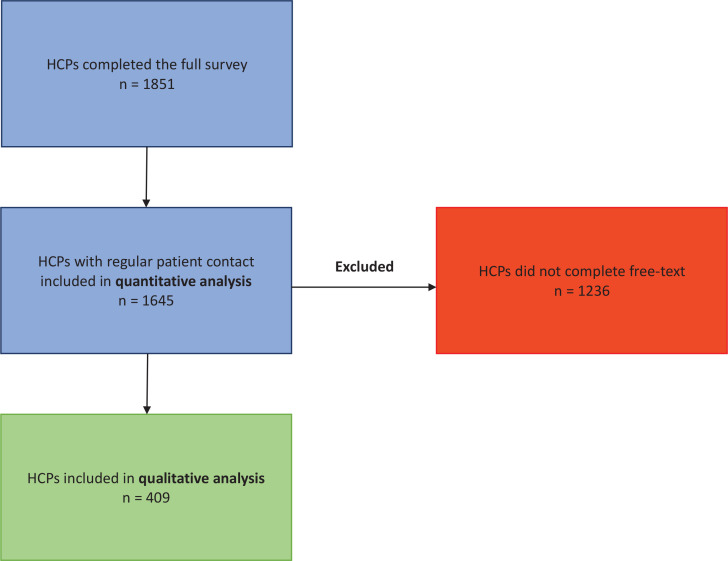
Participant selection flow-chart, cross-sectional online survey performed in 2021 among HCPs in a large secondary care hospital (N=1645)

From June to September 2021, all clinical staff at Lillebaelt Hospital (3530 HCPs in the somatic department and 468 HCPs in the psychiatric department) received the survey link via their work email account, with two reminders sent to non-completers. Characteristics of non-responders are presented elsewhere^15^. The participant selection procedure is shown in Figure 1. An overview of the participant characteristics is given in Table 1. A total of 1851 HCPs completed the entire survey (response rate 46.3%), out of which 1645 (88.9%) responded that they had regular patient contact. These were, therefore, included in the quantitative analysis of this study (‘Total sample’, Table 1). Of the 1645 participants, 409 (24.8%) completed the free-text field in the survey (‘Qualitative subsample’, Table 1), elaborating on factors preventing them from implementing SCS.

**Table 1:** Participant characteristics, cross-sectional online survey performed in 2021 among HCPs in a large secondary care hospital (N=1645)

*Characteristics*	*Qualitative subsample (N=409) n (%)*	*Total sample (N=1645) n (%)*
**Age** (years), mean (SD)	48.4 (11.3)	44.3 (11.8)
**Sex**		
Male	44 (10.8)	219 (13.3)
Female	365 (89.2)	1408 (85.6)
**Healthcare experience** (years), mean (SD)	21.8 (12.0)	17.2 (12.0)
**Department type**		
Somatic	345 (84.4)	1522 (92.5)
Psychiatric	64 (15.6)	123 (7.5)
**Type of clinic**		
Outpatient clinic	113 (27.6)	534 (32.5)
Inpatient bed unit	114 (27.9)	589 (35.8)
A&E/intensive care	71 (17.4)	250 (15.2)
Other	111 (27.1)	221 (13.4)
**HCP category**		
Nurse	260 (63.6)	929 (56.5)
Physician	64 (15.6)	295 (17.9)
Healthcare assistant	29 (7.1)	139 (8.4)
Other*	56 (13.7)	222 (13.5)

* Consists of physiotherapists, occupational therapists, psychologists, social workers, pedagogues, and students. A&E: Accidents and Emergency. HCP: healthcare provider.

### Quantitative results

**Figure 2:**
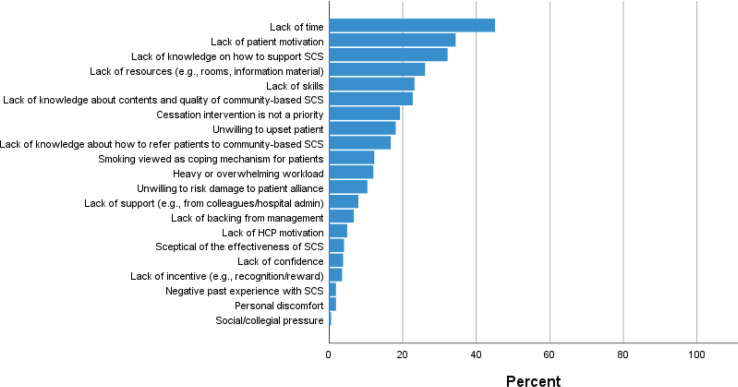
Barriers to smoking cessation support, rank ordered by frequency, cross-sectional online survey performed in 2021 among HCPs in a large secondary care hospital (N=1645)

An overview of the pre-defined barriers in the survey and the frequency by which they were reported by the participants (n=1645) can be found in Figure 2. The most frequently reported barrier to SCS was lack of time, which was reported by 45.1% of the participants. A perceived lack of patient motivation was reported as a barrier by 34.3% of the participants. A lack of knowledge on how to support SCS was reported as a barrier by 32.2%. Lack of resources and lack of SCS skills were reported as barriers by 26.1% and 23.2%, respectively, while lack of knowledge about content and quality of the community-based SCS that patients could be referred to was reported as a barrier by 22.7% of the participants. The remaining barriers were each reported by <20% of the participants.

Supplementary logistic regression analyses were conducted with the purpose of exploring potential predictors of the three most frequently reported barriers (lack of time, lack of patient motivation, and lack of knowledge on how to support). The results of these analyses can be found in Table 2. The regression models showed good fits to the data (χ^2^: 82.90–107.05, p<0.001) and acceptable overall classification accuracies (59.7–66.7%). HCPs with fewer years of experience were slightly more likely to report all three barriers compared to HCPs with more years of experience. HCPs in somatic departments were 66% more likely to report a lack of time as a barrier compared to HCPs in psychiatric departments. Compared to HCPs in outpatient clinics, HCPs in ‘Other’ clinic types (not inpatient bed units and A&E/intensive care) were less likely to report any of the three barriers. Physicians were 2.5 times more likely to report lack of time as a barrier, compared to nurses, and 64% less likely to report lack of knowledge on how to support as a barrier. Compared to nurses, healthcare assistants were 52% more likely to report a lack of patient motivation as a barrier, whereas participants in the ‘Other’ HCP category were 57% less likely to report this barrier.

**Table 2:** Predictors of barriers to providing smoking cessation support, cross-sectional online survey performed in 2021 among HCPs in a large secondary care hospital (N=1645)

*Predictor*	*Lack of time*		*Lack of patient motivation*		*Lack of knowledge on how to support*	
	*OR (95% CI)*	*p*	*OR (95% CI)*	*p*	*OR (95% CI)*	*p*
**Healthcare experience** (years)	0.99 (0.98–1.00)	**0.013**	0.99 (0.98–1.00)	**0.005**	0.97 (0.96–0.98)	**<0.001**
**Department type**						
Psychiatric	-	-	-	-	-	-
Somatic	1.67 (1.11–2.49)	**0.014**	0.99 (0.65–1.50)	0.877	1.22 (0.80–1.85)	0.359
**Type of clinic**						
Outpatient clinic	-	-	-	-	-	-
Inpatient bed unit	0.83 (0.64–1.10)	0.160	0.88 (0.68–1.15)	0.361	1.20 (0.91–1.58)	0.188
A&E/intensive care	0.95 (0.68–1.31)	0.738	0.74 (0.53–1.03)	0.077	1.04 (0.74–1.47)	0.815
Other	0.69 (0.50–1.00)	**0.025**	0.33 (0.22–0.50)	**<0.001**	0.66 (0.45–0.96)	**0.030**
**HCP category**						
Nurse	-	-	-	-	-	-
Physician	2.49 (1.90–3.30)	**<0.001**	1.13 (0.85–1.49)	0.403	0.36 (0.26–0.50)	<0.001
Healthcare assistant	0.69 (0.46–1.00)	0.063	1.52 (1.04–2.23)	**0.032**	0.70 (0.46–1.05)	0.083
Other*	0.78 (0.58–1.10)	0.113	0.43 (0.30–0.61)	**<0.001**	0.97 (0.71–1.32)	0.826

Values of p in bold are statistically significant at the 0.05 level.

* Consists of physiotherapists, occupational therapists, psychologists, social workers, pedagogues, and students. A&E: Accidents and Emergency. HCP: healthcare provider.

### Qualitative results

Based on 409 responses from participants in the thematic analysis, three themes were identified: 1) Concerned about the patient, 2) ‘Not part of my job’, and 3) Inappropriate setting. Themes with associated subthemes are summarized in Table 3.

**Table 3:** Summaries of themes, subthemes, and perspectives, cross-sectional online survey performed in 2021 among HCPs in a large secondary care hospital (N=1645)

Themes	Subthemes	Perspectives
**Concerned about the patient**	Breaking the alliance	Do not want to unnecessarily restrict, offend, or stress the patient, as one does not have a proper relationship with the patient or believes it affects the established relationship or the agenda set by the patient.
	Taking away coping strategy	Smoking is perceived as a factor in quality of life, a tool for de-escalation, or a coping strategy.
	Patient’s autonomy	The perception that patients should decide for themselves and that many do not want to cooperate on the matter.
**‘Not part of my job’**	Others’ responsibility	Think SCS is a responsibility of others, or believe that SCS is too time-consuming for their role.
	Lack of knowledge	Ask for more training and internal guidelines.
**Inappropriate setting**	Work context	As SCS disturbs the agenda, more critical problems must be prioritized first. The surgery and emergency departments are perceived as inappropriate places to initiate SCS. Short contacts leave no option for follow-up, and time is lacking in this work setting.
	Organizational context	Workflow does not encourage SCS, nor does management prioritize it.
	The medical condition of the patient	If patients are severely or acutely affected, it is difficult to address smoking cessation. Concerns that SCS does not change the prognosis of specific diseases or that patients are too affected to integrate it.

*Theme 1: Concerned about the patient*

Under the subtheme of ‘Breaking the alliance’, HCPs were concerned about unnecessarily restricting, offending, or stressing the patient. This was especially the case if the HCPs felt that they had not yet built an alliance with the patient. HCPs were concerned that talking about smoking cessation would affect the already established working alliance negatively or compromise patients’ initiative in bringing their points to the agenda (i.e. shared decision-making and patient-centered communication):

*‘I am afraid that the conversation about smoking cessation will destroy the patient's agenda.’* (Physician, orthopedic surgery)

In the subtheme of ‘Taking away coping strategies’, HCPs described that they perceived smoking to be an important aspect of smokers’ quality of life. They noted that some people use smoking as a way to de-escalate emotive situations or as a coping strategy. It was described that patients had often smoked for many years and that it was now one of the few ‘pleasures’ they had left. The HCPs did not want to take this away from patients, and this, therefore, acted as a barrier to smoking cessation support:

*‘ … to de-escalate the situation and avoid coercion, I must help the patient get some cigarettes.’* (Nurse, psychiatry)

The subtheme of ‘Patient’s autonomy’ included HCPs’ descriptions of their view of smoking as a free choice and something every person should decide for themselves:

*‘I believe it is the patients' own business whether they want to smoke or not.’* (Physician, orthopedic surgery)

HCPs described how this view made them reluctant to talk with patients about smoking and smoking cessation. Moreover, in their experience, some patients are unwilling to cooperate on the matter:

*‘Often, people have “heard it all before” and turn a blind eye to it.’* (Nurse, cardiology)

*Theme 2: ‘Not part of my job’*

Under the subtheme of ‘Others’ responsibility’, HCPs described how they felt that SCS was not part of their job function or specification. Some perceived SCS to be too time-consuming for their role or placed the responsibility with another profession. For example, nurses considered SCS to be the physicians’ responsibility and vice versa:

*‘I am a nurse, and it is the anesthetists who talk with the patients about smoking.’* (Nurse, anesthesiology)

‘Lack of knowledge’ covered HCPs’ expressions of the need for more training and internal guidelines in order to feel comfortable with being responsible for SCS:

*‘I immediately thought it was a doctor's task to refer to municipal smoking cessation. I have never received any training in this …’* (Nurse, A&E)

and

*‘I see it as relevant … but I am unsure if the task is within my professional group … we need internal guidelines for who is responsible.’* (Physiotherapist, medical department)

*Theme 3: Inappropriate setting*

Under the subtheme of ‘Work context’, HCPs expressed that they felt limited by their work setting. Especially in surgery and emergency departments, HCPs pointed out that SCS disturbs the agenda because patients are there for a short period, and it would seem like a lack of situational awareness to ask about smoking habits:

*‘I find it inappropriate in many situations, as patients in the emergency room are there physically for a short time … It seems like lack of situational awareness to ask about smoking habits.’* (Nurse, A&E)

In the subtheme of ‘Organizational context’, some HCPs found that the contextual workflow did not encourage SCS, nor did the management prioritize it. The contextual setup with limited time per patient only allowed for quick contacts, and there were no options for follow-up:

*‘There is no time in the emergency room to dive deep into this topic. Moreover, people come with minor injuries and waiting times, etc., are measured.’* (Nurse, A&E)

Regarding the subtheme of ‘Medical condition of the patient’, HCPs described that they felt it was not appropriate to address smoking and smoking cessation in patients who were severely ill, in acute or terminal phases of an illness. Providing SCS seemed unprofessional as it was considered not to change the prognosis of specific terminal diseases:

*‘It is rarely appropriate to discuss smoking in emergency admissions. Patients cannot decide on smoking cessation during acute illness.’* (Nurse, A&E)

and

*‘I only meet terminal cancer patients, so it makes no sense to encourage smoking cessation.’* (Physician, oncology)

## DISCUSSION

Our study is among the largest surveys, to date, to explore barriers to the implementation of SCS among hospital-based HCPs, based on both quantitative and qualitative evaluation. The most common barriers to SCS identified by the quantitative analysis were lack of time (45.1%), a perceived lack of patient motivation (34.3%), and a lack of knowledge on how to support SCS (32.2%). Also, lack of resources (26.1%), lack of skills (23.2%), and lack of knowledge about the content and quality of community-based smoking cessation programs (22.7%) were reported as barriers by more than one-fifth of the HCPs. These results were also reflected in the qualitative thematic analysis, in which three main themes were revealed: 1) Concerned about the patient, 2)Not part of my job, and 3) Inappropriate setting. HCPs – and most likely also the healthcare organization they work in – perceive smoking cessation support to be an optional extra service. While this, to some extent, might be a valid point in certain specific specialties, such as during surgery and in acute care units, in most diseases smoking cessation can have a profound impact on illness progression and treatment outcomes^2,5,19,20^. Supplementary results of our study indicate that, compared to nurses, physicians are more likely to report lack of time as a barrier. This result might mirror a classical labor division, where physicians give brief advice and guidance while nurses engage in conversations and care management^21^. In the somatic setting, HCPs were 66% more likely to report lack of time as a barrier compared to the psychiatric setting.

Hence, patient groups with smoking-related diseases, such as lung cancer, chronic obstructive pulmonary disease, and cardiovascular disease, are potentially being deprived of an efficacious treatment service when treated at the hospital^22^. The themes identified in the qualitative analysis illustrate that – from the HCP’s viewpoint – barriers to implementation may arise at multiple levels of healthcare delivery: the patient level (‘Concerned about the patient’), the provider level (‘Not part of my job’), and the organizational level (‘Inappropriate setting’).

### Healthcare professionals’ perceived barriers at the patient level

At the patient level, the main barriers were breaking the alliance with the patient, taking away coping strategies, and harming quality of life. Other barriers raised were bad experiences with SCS or a lack of patient cooperation and motivation. For example, in the quantitative analysis, a lack of patient motivation was perceived to be a barrier for more than one-third of HCPs. Healthcare assistants were more likely than nurses to report this barrier, which could potentially be explained by the different work tasks and, thereby, the different levels of insight into patients’ inner factors between these types of professions.

A study by Russel et al.^23^ investigated the barriers faced by HCPs in providing SCS in Australia. The study highlights similar main themes: the clinical setting, knowledge, consistency, and appropriateness. The theme of appropriateness reflects some of the same points and concerns as the barriers identified at the patient level in the present study. They point out challenges to initiating SCS in specific groups of patients, e.g. patients in palliative care, and that some HCPs express empathy for patients who smoke, as quitting can be a major frustration and very stressful. They also address safety issues in relation to HCPs’ concerns that aggressiveness in patients could be a barrier.

Naturally, HCPs often meet patients undergoing significant crises due to serious illness, losing family members, suicidal impulses, etc. As part of assessment and treatment, HCPs are trained to ask targeted questions that, in a non-healthcare setting, often would be considered private. A study conducted in 2023 by Malhotra et al.^24^ reports the most common barrier to advance care planning conversations with patients to be ‘lack of time’, whereas ‘upsetting the patient’ was rated as the least important barrier. Therefore, it can appear counterintuitive that HCPs could consider conversations about smoking and smoking cessation as interfering with patients’ limits and damaging the patient alliance.

Further studies on patient perspectives find that patients expect HCPs to offer them SCS^25,26^. Studies such as those might encourage HCPs to break the personal limits that our present study highlights.

### Healthcare professionals’ perceived barriers at the provider level

The main perspective concerning the provider level was a lack of division of responsibilities. Most HCPs expressed that SCS would be better delivered elsewhere, believed it was too time-consuming for them, or said that they lacked knowledge and internal guidelines. HCPs with fewer years of experience were more likely to report all three of the most frequent barriers in this study: lack of time, lack of patient motivation, and lack of knowledge on how to support. Especially the relatively inexperienced could, therefore, be in need of clear guidelines and policies to guide their practice in this area.

Russel et al.^23^ also reflect these areas in the two themes ‘clinical setting’ and ‘knowledge’, in which barriers such as lack of capacity, policies, and training needs, are highlighted. In their third theme, ‘consistency’, they discuss whether SCS should be acknowledged as ‘everyone’s responsibility’, as it comes down to what the patient picks up from each interaction with HCPs.

It appears that a systematic approach to overcoming these personal barriers among HCPs is needed. For example, in a study by Michie et al.^27^, behavior change interventions to overcome barriers are described using the ‘COM-B-system’. The system is a ‘behavior system’ that helps us understand behavior by way of three components known to generate behavior: capability, opportunity, and motivation (the COM-B system). According to the authors, these components interact to create behavior and form a hub of the ‘behavior change wheel’ (BCW). Importantly, the elements can influence one another. For example, opportunity can affect motivation, as can capability.

Some HCPs in the present study believed SCS to be a long and comprehensive process and did not feel they had the time or capability to implement it. Indeed, SCS can be a complex intervention that includes multiple components, such as: 1) assessment of smoking, 2) providing information and advice, 3) planning and referral to further support, and 4) follow-up on smoking cessation attempts. However, when HCPs are trained in the approach, such conversations can be relatively limited in time and can have a significant impact on patients’ smoking cessation rates. This is illustrated in a meta-analysis, in which ‘motivational interviewing’ with a duration as short as 10 minutes increased the chances of smoking cessation by 26%, compared to brief advice or usual care^28^.

Lack of knowledge about how to manage SCS, the clinical (disease-related) benefits of smoking cessation, and its expected extent, are important findings that can inform and support training in and implementation of SCS in daily work at a hospital and at the organizational level. In our study, physicians were less likely to report a lack of knowledge as a barrier compared to nurses, but it is unclear whether this finding genuinely reflects sufficient levels of knowledge or a self-perceived level of knowledge that aligns with the low levels of support that are delivered in practice^15^.

### Healthcare professionals’ perceived barriers at the organizational level

At the organizational level, our results show that many HCPs did not consider that their workflow was compatible with SCS, as there was a lack of time, and the setting lacked the option to follow up. A high-volume workload leading to less capacity, context appropriations, and lack of resources, is also pointed out by Russel et al.^23^.

It can be seen as a paradox that HCPs find it unprofessional to address smoking habits in patients who are severely ill or screened for a severe illness, despite the fact that several studies show a beneficial effect of addressing smoking habits and initiating SCS in exactly these periods of a patient’s illness trajectory^29,30^.

Even though the clinical benefits of implementing cessation services in hospital settings have been recognized for decades, the organizational barriers perceived by HCPs in the present study illustrate a need for systematic integration at an organizational level. According to Pipe et al.^31^, the Ottawa Model for Smoking Cessation (OMSC) developed at the University of Ottawa Heart Institute can be used for this purpose. OMSC consists of multiple components that can support the implementation of a smoking cessation intervention in healthcare organizations. The intervention components include training for staff in evidence-based tobacco treatment, provision of tools and resources, digital and telephone follow-up scripts, materials to complement face-to-face contact, and, notably, materials to support performance evaluation.

Lastly, if performance indicators at the organizational level do not include SCS, stakeholders, leaders, and staff have no way of tracking progress, and interventions can thereby more easily be put aside in favor of other interventions that are monitored regularly^32^.

Most Danish hospitals have sought to implement Very Brief Advice (VBA)^14^ to support all smokers, regardless of their motivation to quit. Nonetheless, the results of our former study^15^ reveal that more than 25% of the included HCPs never or rarely ask patients about their smoking status.

### Strengths and limitations

The present study is one of the first to explore barriers to the implementation of smoking cessation among HCPs across all departments at a large hospital. It includes a large sample of employees who participated anonymously, thereby increasing the reliability and reducing bias in the results. A relatively large number of participants gave a subjective account of their perceived barriers to SCS, allowing for both breadth and depth in the qualitative data set.

Some limitations should be noted. First, participants were prompted to give their free-text account as an addition to the pre-defined list of barriers. We can, therefore, not present in-depth accounts of individual participants’ subjective understanding of each barrier. An interview-based approach could have yielded a deeper understanding of barriers from the perspective of individual HCPs, but it would also have compromised the sample size of the study. Second, some response options in the pre-defined list of barriers in the survey overlapped, e.g. ‘lack of skills’ and ‘lack of knowledge’. Third, HCPs’ own smoking status has previously been shown to influence both their perceived relevance of smoking cessation for patients and their engagement in SCS^33,34^. However, we did not assess participants’ smoking status in the present study, as that could potentially have negatively affected their willingness to take part in the survey. Fourth, only 24% of the total sample gave a free-text response in the survey. However, the characteristics of the qualitative subsample were generally comparable to those of the total sample. Fifth, the ‘Other’ category was a common predictor of the most frequent barriers in the present study, but as the participants were not given the opportunity to describe these categories further, it was not possible to conclude exactly what types of HCPs and clinics these categories include. Sixth, data were only collected from the HCPs’ perspective, not including the patient and/or management perspective, which limits the external validity of the results. Future studies should use triangulation approaches to data collection by addressing patient, provider, and organizational perspectives.

## CONCLUSIONS

The combination of quantitative and qualitative analyses in this study highlights barriers to SCS on multiple levels among HCPs in a hospital setting: the patient, provider, and organizational level. Therefore, with the objective of providing more efficient smoking cessation support to patients in the hospital setting, barriers at all levels should be addressed when designing implementation strategies. Future studies and implementation should include data from the patient’s perspective, with the aim of increasing the validity of the findings.

## Supplementary Material



## Data Availability

The data supporting this research cannot be made available for privacy or other reasons.
