# Supplementary figures and images for: Barriers to implementation of smoking cessation support among healthcare professionals in the secondary healthcare sector: A qualitative and quantitative evaluation
Source: Tob Prev Cessat. 2024 Feb 22;10:10.18332/tpc/183775. doi: 10.18332/tpc/183775 (PMC10882562; doi:10.18332/tpc/183775)

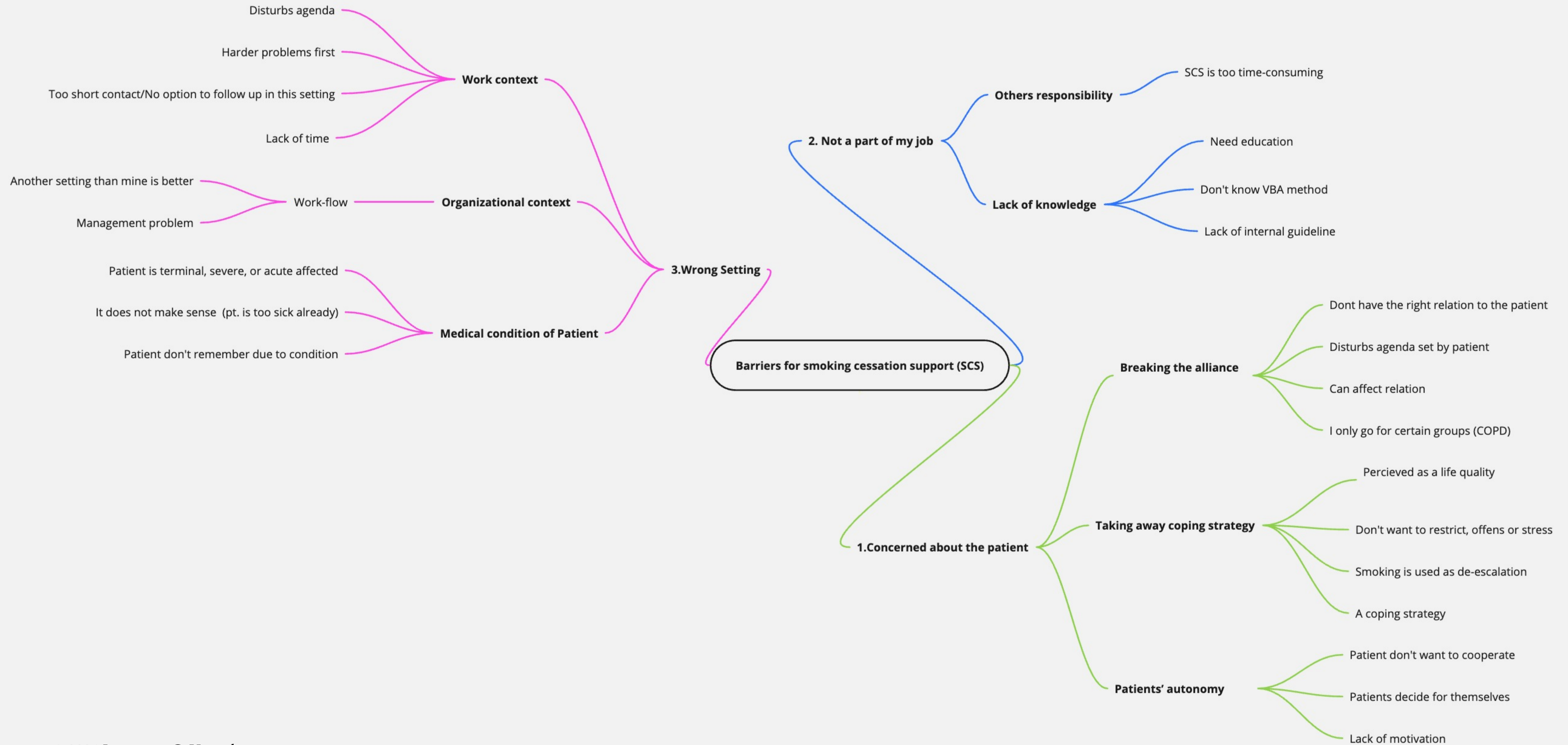

Supplement: Supplementary file 1 [file TPC-10-12-s1.pdf]
